# Plasma Fibroblast Growth Factor 21 Is Associated with Subsequent Growth in a Cohort of Underweight Children in Bangladesh

**DOI:** 10.1093/cdn/nzz024

**Published:** 2019-03-30

**Authors:** Michael B Arndt, Barbra A Richardson, Mustafa Mahfuz, Tahmeed Ahmed, Rashidul Haque, Md Amran Gazi, Grace C John-Stewart, Donna M Denno, Jarrad M Scarlett, Judd L Walson, coordination with The Interactions of Malnutrition & Enteric Infections: Consequences for Child Health and Development Project Network

**Affiliations:** 1 Department of Epidemiology, Seattle, WA; 2 Department of Global Health, Seattle, WA; 3 Department of Biostatistics, Seattle, WA; 4 Department of Medicine, Seattle, WA; 5 Department of Pediatrics, University of Washington, Seattle, WA; 6 PATH, Seattle, WA, Dhaka, Bangladesh; 7 Nutrition and Clinical Services Division, International Centre for Diarrhoeal Disease Research, Bangladesh (icddr,b), Dhaka, Bangladesh; 8 Parasitology Laboratory, icddr,b, Dhaka, Bangladesh; 9 Childhood Acute Illness and Nutrition Network, Nairobi, Kenya; 10 Department of Pediatric Gastroenterology and Hepatology, Seattle Children's Hospital, Seattle, WA

**Keywords:** FGF21, growth hormone resistance, protein deficiency, stunting, supplementation, undernutrition, underweight

## Abstract

**Background:**

Current nutritional intervention strategies have not proven effective in improving childhood ponderal and linear growth in underweight and stunted children. Novel markers are needed to classify children who are likely to respond to available interventions and to identify those requiring additional interventions. Fibroblast Growth Factor 21 (FGF21), an endocrine hormone that regulates metabolism and growth during periods of reduced protein intake, may be useful in this context.

**Objectives:**

We aimed to determine the associations between plasma FGF21 concentrations and subsequent growth, and the association between change in FGF21 concentrations and concurrent growth, in children receiving nutritional supplementation.

**Methods:**

A total of 120 children between ages 6 and 13 mo with weight-for-age *z* score (WAZ) between −3 and −2 were enrolled from an urban slum in Dhaka, Bangladesh. Children received 376-kcal feeding supplements daily for 5 mo and were followed for 5 additional mo. FGF21 was measured in plasma collected at enrollment and month 5. FGF21 values that fell above the 90th percentile of baseline concentrations (1056.5 pg/mL) were considered high. Linear regression was used to examine the association between baseline FGF21 status and 5-mo change in WAZ and length-for-age *z* score (LAZ), and the association between 5-mo change in FGF21 and concurrent WAZ and LAZ change.

**Results:**

The median baseline FGF21 concentration was 241.4 pg/mL (IQR: 111.7, 451.3 pg/mL). On average, children with high baseline FGF21 gained 0.58 WAZ (95% CI: 0.28, 0.88) and 0.54 LAZ (95% CI: 0.23, 0.84) more during supplementation than those with low values. Change in FGF21 concentration during supplementation was negatively associated with change in WAZ (−0.48; 95% CI: −0.67, −0.29) and LAZ (−0.31; 95% CI: −0.52, −0.11).

**Conclusions:**

FGF21 may be a useful marker of growth faltering and may allow identification of children who are more or less likely to respond to nutritional supplementation. This trial was registered at clinicaltrials.gov as NCT02441426.

## Introduction

Poor child growth contributes substantially to morbidity and mortality in resource-limited countries (1, 2). Poor linear growth, marked by stunting [length-for-age *z* score (LAZ) < −2],

is reflective of the cumulative impacts of chronic deprivation and repeated infectious insults. Poor weight gain, marked by underweight [weight-for-age *z* score (WAZ) < −2] and wasting [weight-for-length *z* score (WLZ) < −2], may indicate acute deprivation or illness (3). Nutritional interventions alone often do not effectively reverse poor childhood growth, particularly stunting (4). There is a need for markers to identify children who are likely to grow while receiving supplemental nutrition as well as those in need of additional interventions targeting other causes of malnutrition such as chronic or recurrent infections and/or poor gut health and function.

Hormonal responses to nutrient intake may correlate with a child's growth pattern and may be useful as markers of growth responsiveness to intervention. Growth hormone (GH) is the principal endocrine regulator of growth and is secreted nightly by the anterior pituitary (5). GH regulates growth in part by binding to receptors in the liver that control expression of insulin-like-growth factor I (IGF-I), one of the hormones responsible for stimulating growth in local tissues and systemically. GH resistance, defined by elevated GH concentrations and low concentrations of IGF-I, is exhibited in states of undernutrition, including chronic caloric insufficiency, protein deficiency, and isolated micronutrient deficiencies (zinc, vitamin A, magnesium), and has been associated with systemic inflammation (6, 7). GH resistance may mediate the relation between these nutritional deficiencies and poor linear and ponderal growth in children. Because GH resistance is not nutrient specific, IGF-I and GH cannot be used to identify children who are likely to grow in response to supplemental nutrition. An endocrine hormone called Fibroblast Growth Factor 21 (FGF21) may be more informative, because it appears to play a role in the pathway by which chronic caloric/protein deprivation in particular triggers GH resistance and inhibits skeletal growth (6, 8–13).

Fibroblast growth factors are a family of protein growth factors that regulate diverse biological processes including growth and development. FGF21 is an endocrine hormone produced primarily by the liver and adipocytes that regulates glucose and lipid metabolism, and is a signal of protein restriction that regulates metabolism and growth during periods of reduced protein intake (8, 9, 14). In humans, FGF21 concentrations rise after sustained fasting and in response to protein deprivation (14–16). Chronic exposure to FGF21 results in reduced expression of hepatic GH receptors, inhibition of GH signaling, and disruption of GH action at the bone growth plate (6, 8–13). Limited pediatric data suggest that serum and plasma concentrations of FGF21 are negatively associated with linear growth in healthy and preterm children younger than 1 y (13, 17). Collectively, these data suggest that FGF21 may be a useful marker in identifying undernourished children whose growth is impeded by insufficient macronutrient (protein) intake. Therefore, high concentrations of FGF21 may identify those children who are more likely to grow in response to nutritional supplementation than others whose poor growth is attributable to other factors, such as environmental enteropathy, a subclinical intestinal disorder prevalent among children in resource-limited settings which has been associated with linear growth faltering, reduced oral vaccine immunogenicity, and cognitive impairment (18–20).

The present study examines the association between circulating concentrations of FGF21 and subsequent growth in a cohort of underweight children receiving nutritional supplements in urban Bangladesh.

Study protocols for The Interactions of Malnutrition & Enteric Infections: Consequences for Child Health and Development (MAL-ED) multisite “Case Control” study were reviewed and approved by the Institutional Review Boards of all collaborating institutions, and governmental and regional health authorities, including the Ethical Review Committee (ERC) of the International Centre for Diarrhoeal Disease Research, Bangladesh (icddr,b) in Bangladesh (21, 22). Informed consent was taken from the legal guardians of the children before enrollment. Caregivers were not compensated for participating in the research; however, transportation costs and wage loss were covered if required for participation. The present study, including the quantification of FGF21 in previously collected samples, was not part of the initially planned MAL-ED analysis, and a separate proposal was approved by the ERC. The University of Washington Institutional Review Board approved the use of coded data and samples previously gathered in icddr,b's ERC-approved study.

## Methods

### Study population

This prospective cohort study utilized data from a subsample of 120 children enrolled in the MAL-ED Case Control study, a 1-y prospective interventional study (NCT02441426). In the parent study, 500 moderately to severely underweight (WAZ < −2) children aged 6–23 mo living in the Bauniabadh area of section 11 of Mirpur, an urban slum in Dhaka, Bangladesh, were recruited by field workers at a child feeding center from February, 2009 through April, 2011 (21, 23). Children were identified either through an initial community-wide WAZ screening of children aged 6–24 mo or by active surveillance at the feeding center, which continued through the study period.

The sample of 120 children was chosen for the present analysis based on an a priori sample size calculation for a simple linear regression of 6-mo change in FGF21 on concurrent change in LAZ. This sample size provided 80% power to detect true slopes of −0.004 or 0.004 for the line obtained by regressing 6-mo change in LAZ against concurrent change in FGF21 with an α level of 0.05, based on SDs of 0.56 LAZ and 37.3 pg/mL and a correlation coefficient of −0.19 (see **Supplemental Methods** for detail) (17).

**FIGURE 1:**
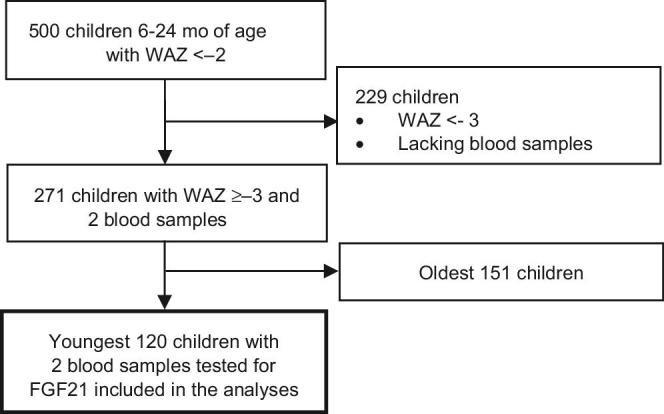
A total of 500 children 6–24 mo of age were enrolled in the parent study. Children in this analysis were those with a WAZ > −3 and plasma samples available from enrollment and month 5 of follow-up. The youngest 120 children meeting these criteria were included. FGF21, Fibroblast Growth Factor 21; WAZ, weight-for-age *z* score.

The sample was restricted to children who were moderately underweight at enrollment (WAZ > −3, but <−2) and had ≥200 μL of stored plasma available from enrollment and month 5 of follow-up (**Figure 1**). In order to limit potential age-related heterogeneity of the FGF21 assay results, the youngest 120 children of the parent study were included in this sample. These 120 children were between 6 and 13 mo of age at enrollment. Exclusion criteria included illnesses that might affect nutritional status or response to treatment (e.g., severe diarrhea or pneumonia at the time of enrollment; persistent diarrhea; cleft lip or palate; blindness; tuberculosis; jaundice; renal or cardiac disease; cerebral palsy; and chromosomal disorders, including trisomy 21). Details of the parent study were published previously (21, 22).

Each participant received 376 kcal of locally produced “Pushti packet” food supplements daily for 5 mo, or until the child achieved a WAZ ≥ −1. Study staff directly observed consumption of Pushti supplements at the study clinic (23). Each 188-kcal Pushti packet contained 20 g roasted rice powder, 10 g roasted lentil powder, 3 g soybean oil, and 5 g molasses (24). All children also received high-dose vitamin A capsules at 6-mo intervals and routine immunizations, and their caregivers received counseling on how to improve the child's nutritional status (21). Enrolled children initially received 1 sachet of MoniMix micronutrient powder (Renata Limited) daily for 2 mo (23). Each sachet contained 12.5 mg Fe, 5 mg Zn, 300 µg vitamin A, 150 µg folic acid, and 50 mg vitamin C. Children enrolled after August, 2010 received packets daily for 4 mo. All children who developed severe acute malnutrition (SAM; WLZ < −3 and/or bipedal edema) were admitted to the icddr,b Dhaka hospital and treated following current WHO guidelines (25). Children with SAM were not released for community-based care until WLZ exceeded −2. Ten children (8.3%) met the criteria for SAM at enrollment or at least once within the 5-mo supplementation period, and 4 children (3.3%) developed SAM within the 5-mo postsupplementation period.

### Data collection

Household/maternal characteristics were assessed at enrollment, and caregivers reported morbidity (e.g., diarrhea, respiratory disease, or fever) and child diet twice a week to study staff during household visits. Child anthropometry was assessed at monthly intervals. Children were weighed using pediatric balances with a certified accuracy of 100 g; length was measured using a marked platform with a sliding footboard (26).

Blood samples were gathered in lithium heparin–coated tubes for the purpose of micronutrient testing (zinc, retinol, vitamin D, and ferritin) at enrollment and after the 5 mo of nutritional supplementation, and were centrifuged at 2000 × *g* for 10–15 min. Storage temperatures of –70°C apply for any aliquots that were not tested immediately. Environmental enteropathy was assessed in children at enrollment using α-1-antitrypsin (AAT), a serum protein whose fecal excretion is elevated in states of increased intestinal permeability (27, 28). Stool samples were collected by caregivers and stored in cold packs within 1 h without fixative before collection by a field worker during a home visit. Stool samples were frozen at −70°C pending processing (29). Stool samples were excluded from children who had diarrhea symptoms within 7 d before collection and from those who had dual sugar permeability testing within 1 d before collection. These factors alter stool water content to a variable extent, making the interpretation of AAT concentrations unreliable without measuring dry weights.

### Laboratory methods

All procedures were conducted in the icddr,b laboratories in Dhaka. Plasma retinol was quantified using HPLC (30). An aliquot of plasma was deproteinized with methanol containing 50 μg% and retinol was extracted into hexane. The hexane layer was transferred to a clean vial, evaporated under nitrogen, re-dissolved in mobile phase (95% methanol), and injected into an HPLC column. Three plasma pool samples with assigned values set against standard serum from the National Institute of Science and Technology were run with each set of samples, and the concentration of retinol was calculated based on the known concentration of retinol in the pool samples. Whole blood was collected in microcuvettes and quantitative determination of hemoglobin was performed using the HemoCue HB201 analyzer. FGF21 was quantified in plasma samples using a commercially available ELISA (R&D Systems) per package insert instructions. Sulfuric acid (9.8%) stop solution was produced locally. Each plasma sample was tested in duplicate, read directly at 450 nm using Gen5 software (BioTek), and results were expressed as the mean value. A wavelength correction was not performed, as neither a 540-nm nor a 570-nm filter was available. The Gen5 software was programmed to extrapolate concentrations for samples whose absorbance exceeded the highest standard (2000 pg/mL) by <20%, or fell below the lowest standard (31.3 pg/mL) by <20%. In cases in which the sample was out of range, the software reported that the concentration was either >2462 pg/mL or <25.426 pg/mL; and therefore, such results were assigned a value of 2462 pg/mL or 25.4 pg/mL, respectively. ELISA results from 2 of the 120 enrollment samples exceeded the absorbance of the highest standard (in both wells), and results from 4 samples fell below the absorbance of the lowest standard. At month 5, ELISA results from 2 of the 120 samples exceeded the absorbance of the highest standard, and results for 6 samples were less than the absorbance of the lowest standard.

AAT was measured in stool samples using commercially available ELISA kits (BioVendor), and was run per package insert instructions at a dilution of 1:500 (29, 31). Samples out of range of the standard curve for the AAT assay were run at higher or lower concentrations (as appropriate) (31).

### Statistical methods

Analyses were performed using STATA version 14.2 SE (StataCorp). The WHO's Anthro software was used to calculate *z* scores (LAZ and WAZ) from raw anthropometric data using standards established in the 2006 WHO Multicentre Growth Reference Study Group. Height observations were dropped from the data set if deemed implausible (≥1 cm lost from the previous month or bounded by values lower by ≥1 cm). Implausible height data were omitted from 1 child at 2 time points (enrollment and month 1), and from 7 children at single time points in the intervention period (1.3% of measurements within that period).

The distributions of anthropometric measurements, plasma biomarker concentrations, and fecal AAT concentrations were evaluated for normality using Shapiro–Wilk tests. Pearson correlations were calculated for baseline plasma FGF21 concentrations with fecal AAT, plasma micronutrient concentrations (ferritin, vitamin D, zinc, retinol), blood hemoglobin, and plasma concentrations of the systemic inflammation markers α-1-glycoprotein (AGP) and C-reactive protein (CRP). A series of regression models was used to test whether FGF21 was associated with growth during nutritional supplementation, with plasma FGF21 as a continuous (concentration in nanograms per milliliter for ease of interpretation) or a binary (high compared with low) variable.

Student's *t* tests with unequal variances were used to compare the mean baseline FGF21 concentrations between children whose LAZ increased and those whose LAZ decreased over the 5-mo supplementation period. The comparison was also done for WAZ. Linear regression was used to test whether baseline FGF21 concentration was associated with improvements in nutritional status (change in LAZ or WAZ) over the 5-mo supplementation period. FGF21 was modeled in units of nanograms per milliliter rather than picograms per milliliter to ease the interpretability of the model coefficients. Models included terms to adjust for the pertinent baseline anthropometric measurement and plasma retinol and ferritin concentrations (considering the co-administration of micronutrient supplements).

**FIGURE 2:**
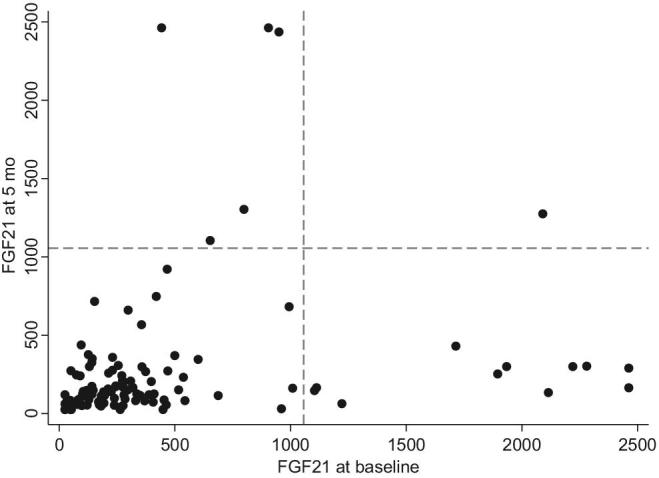
Scatterplot of plasma FGF21 concentrations at enrollment and after supplementation. Dashed line indicates the 90th percentile of baseline values (1056.5 pg/mL). FGF21, Fibroblast Growth Factor 21.

Examination of the baseline FGF21 concentrations revealed a distribution that was heavily skewed (**Figure 2**). As there was interest in analyzing differences between the groups of children with extreme FGF21 values and those without, children were grouped by FGF21 above (high) or below (low) the 90th percentile of the baseline concentration data (1056.5 pg/mL). This cutoff was based on the pre–nutritional supplementation FGF21 concentration data rather than the pooled data, because the baseline FGF21 distribution in the sample was likely more representative of the population of underweight children living in this setting. To assess potential confounders, Student's *t* tests with unequal variances were used to compare the mean baseline plasma micronutrient concentrations, plasma AGP concentrations, fecal AAT concentrations, and anthropometry in children with and without high FGF21 at enrollment.

Student's *t* tests with unequal variances were used to compare the mean change in LAZ and WAZ scores in groups of children whose plasma FGF21 concentration increased or decreased over the 5-mo supplementation period. Linear regression was used to test whether baseline FGF21 status (high vs. low) was associated with improvements in nutritional status over the 5-mo supplementation period. Models were adjusted for baseline anthropometric measurements and micronutrients found to be unbalanced at baseline based on 2-sided *t* tests (ferritin *P* = 0.0339, retinol *P* = 0.0022).

Lowess curves were used to describe and compare the 10-mo WAZ and LAZ trajectories of children who had high or low plasma FGF21 at baseline.

To determine whether the relation between FGF21 status and subsequent growth was the same in the supplementation and postsupplementation periods, a linear mixed-effects model was used to model 5-mo change in WAZ and LAZ. Each model contained a random intercept term, time-varying terms for period (supplemental compared with post), and the following measures at the start of the period: high FGF21, plasma retinol concentration, and plasma ferritin concentration. An interaction term between high FGF21 and period was used to test whether an association between FGF21 status and 5-mo change in *z* score growth differed between baseline measurements and those taken immediately after supplementation. If the interaction term was significant, then a linear regression model was created for 5-mo change in that growth parameter for the postsupplementation period.

To determine whether children with high compared with low baseline FGF21 concentrations exhibited different growth velocities on a fine scale during supplementation, linear mixed-effects models with first-order autoregressive residual structures were used to model monthly LAZ and WAZ. Children were excluded from a model if they contributed <2 anthropometric observations during the time period. Each model contained a random intercept term, fixed terms for high baseline FGF21 and month of follow-up, and an interaction term for the “effect” of high baseline FGF21 on monthly change in *z* score. The interaction term was used to assess whether monthly change in *z* score differed by baseline FGF21 status. If an interaction term was significant, the models for that growth parameter were stratified by FGF21 status.

Finally, linear regression was used to test whether change in FGF21 concentration was associated with concurrent change in LAZ or WAZ over the 5-mo supplementation period. Change in FGF21 was modeled in units of nanograms per milliliter rather than picograms per milliliter to ease the interpretability of the model coefficients. Models included terms to adjust for baseline values of the pertinent anthropometric measurement and baseline plasma concentrations of FGF21, retinol, and ferritin.

## Results

A total of 120 moderately undernourished children with baseline and month 5 blood samples were included from the parent study (Figure 1). Age at enrollment ranged from 182 to 385 d and 56% of subjects were female (**Table 1**).

**TABLE 1:** Characteristics of participants and their mothers^1^

	*n* (%) or median [IQR]
Children	
Female	67 (55.8)
Age at enrollment, d	278 [221–337]
Anthropometry at enrollment^2^	
LAZ	−2.17 [−2.65 to −1.60]
Stunted	71 (59.1)
WAZ	−2.41 [−2.74 to −2.22]
WLZ	−1.70 [−2.16 to −1.27]
Wasted	37 (31.1)
Anthropometry (month 5)^3^	
LAZ	−2.58 [−3.00 to −2.02]
Stunted	88 (75.9)
WAZ	−2.49 [−2.81 to −2.17]
WLZ	−1.60 [−2.04 to −1.11]
Wasted	31 (27.0)
Anthropometry (month 10)^4^	
LAZ	−2.71 [−3.15 to −2.29]
Stunted	87 (86.1)
WAZ	−2.52 [−2.87 to −2.12]
WLZ	−1.58 [−2.06 to −1.15]
Wasted	28 (27.7)
FGF21, pg/mL	
Baseline	241.4 [111.7–451.3]
Five months	139.1 [78.2–272.0]
AAT,^5^ mg/g	
Baseline	0.268 [0.163–0.673]
Five months	0.336 [0.163–0.695]
Mothers	
Age, y	23 [20–27]
Marital status	
Married: only wife	102 (85.0)
Married: polygamous	17 (14.2)
Age at first marriage	17 [15–18.5]
Maternal education	
Never attended school	22 (18.3)
Schooling completed, y	4 [2–6]

^1^ *n* = 120 unless otherwise noted. AAT, α-1-antitrypsin; FGF21, Fibroblast Growth Factor 21; LAZ, length-for-age *z* score; WAZ, weight-for-age *z* score; WLZ, weight-for-length *z* score.

^2^ *n* = 119 children with valid LAZs at enrollment.

^3^ *n* = 115 children with valid anthropometry from month 5 (116 with valid WAZs).

^4^ *n* = 101 children with valid anthropometry from month 10.

^5^ *n* = 102 children with valid AAT measurement.

### Maternal demographics

The median age of enrolled mothers was 23 y; most were married and monogamous (85%) and had married in their mid to late teens (Table 1). More than 80% of mothers had received some schooling, with most completing ≥4 y. The majority of mothers had been pregnant previously (median age of first pregnancy: 18 y). The median monthly household income was 7000 Bangladeshi Taka (IQR: 5000–9000), equivalent to US$90.

### Child anthropometry

Valid anthropometry data were available from 3–5 monthly time points during the 5 mo of supplementation, including 1 child who ceased supplementation at month 4 owing to WAZ graduation. In all, 101 children had valid anthropometry data available 5 mo after cessation of supplementation (month 10).

At enrollment, nearly 60% of children were stunted and >30% were wasted (Table 1). Median LAZ and WAZ were –2.2 and –2.4, respectively. Median LAZ declined to –2.7 after 10 mo of follow-up, whereas WAZ remained relatively unchanged at –2.5. After 10 mo of follow-up, 86% were stunted and 28% were wasted.

### Plasma FGF21 distribution, categorization, and cofactors

The median FGF21 concentration among the 120 underweight children was 241 pg/mL at enrollment and 139 pg/mL at the month 5 follow-up visit (Table 1). At enrollment, plasma FGF21 was correlated with plasma retinol (ρ = −0.3365, *P* = 0.0002) and AGP (ρ = 0.2657, *P* = 0.0039) concentrations (**Supplemental Table 1**). After supplementation (month 5), FGF21 was still correlated with plasma retinol (ρ = −0.2422, *P* = 0.0088) and AGP (ρ = 0.2779, *P* = 0.0028) concentrations (Supplemental Table 1).

Baseline LAZ, WLZ, plasma AGP concentrations, and blood hemoglobin concentrations were normally distributed based on nonsignificant Shapiro–Wilk tests. Baseline WAZ, fecal AAT concentration, and plasma concentrations of FGF21, ferritin, retinol, vitamin D, zinc, and CRP were nonnormally distributed based on significant Shapiro–Wilk tests. LAZ, WAZ, WLZ, and plasma concentrations of zinc were normally distributed after 5 mo of supplementation based on nonsignificant Shapiro–Wilk tests. Fecal AAT concentration, and plasma concentrations of FGF21, ferritin, hemoglobin, retinol, vitamin D, AGP, and CRP were nonnormally distributed after 5 mo of supplementation based on significant Shapiro–Wilk tests.

As described in the Methods section, high FGF21 was defined as values that exceeded the 90th percentile of baseline FGF21 concentrations (>1056.5 pg/mL). Eight (66.7%) of the 12 children with high baseline FGF21 were female. On average, children in the high baseline FGF21 group had poorer growth status, more systemic inflammation (median plasma AGP), and lower median plasma ferritin and retinol concentrations at enrollment than those in the low baseline FGF21 group (**Supplemental Table 2**).

In total, 6 children (5%) had high FGF21 at the end of supplementation; all but 1 were different children from those who had high values at baseline. Children with high FGF21 after supplementation had significantly lower concurrent mean plasma retinol (23.7 compared with 17.7 µg/dL, *P* = 0.0055) and lower concurrent mean plasma CRP (1.6 compared with 4.7 mg/dL, *P* = 0.0192) than other children. Significant differences in other micronutrients and inflammatory markers were not observed postsupplementation.

### FGF21 and weight gain

There was no significant difference in mean baseline plasma FGF21 concentration in children who gained WAZ (*n* = 60) compared with those who lost WAZ (*n* = 56) over the 5 mo of supplementation (488.4 compared with 381.3 pg/mL, *P* = 0.2986). Plasma FGF21 concentrations at enrollment were not significantly associated with 5-mo change in WAZ during supplementation (**Table 2**); however, high FGF21 concentrations (>90th percentile) were significantly associated with 5-mo change in WAZ. Children with high FGF21 at enrollment gained a mean 0.58 WAZ more during supplementation than those with low FGF21 at enrollment (95% CI: 0.28, 0.88).

**TABLE 2:** Multiple linear regression models of the relation between baseline plasma FGF21 concentration or FGF21 status (high compared with low) and 5-mo change in WAZ and LAZ^1^

	Coefficient	(95% CI)
Plasma FGF21 concentration		
ΔWAZ		
WAZ^2^	−0.055	(−0.334, 0.224)
FGF21,^2^ ng/mL	0.213	(−0.003, 0.428)
Ferritin,^2^ μg/L	0.001	(−0.002, 0.003)
Retinol,^2^ μg/dL	0.011	(−0.003, 0.024)
Constant	−0.483	(−1.301, 0.335)
ΔLAZ		
LAZ^2^	−0.323***	(−0.465, −0.181)
FGF21,^2^ ng/mL	0.194*	(0.025, 0.363)
Ferritin,^2^ μg/L	−0.001	(−0.003, 0.002)
Retinol,^2^ μg/dL	0.009	(−0.002, 0.021)
Constant	−1.356***	(−1.841, −0.871)
FGF21 status (high vs. low)		
ΔWAZ		
WAZ^2^	−0.031	(−0.323, 0.261)
High FGF21^2^	0.581***	(0.278, 0.884)
Ferritin,^2^ μg/L	0.001	(−0.001, 0.003)
Retinol,^2^ μg/dL	0.013*	(0.000, 0.026)
Constant	−0.441	(−1.238, 0.357)
ΔLAZ		
LAZ^2^	−0.316***	(−0.454, −0.177)
High FGF21^2^	0.535**	(0.233, 0.836)
Ferritin,^2^ μg/L	−0.001	(−0.003, 0.002)
Retinol,^2^ μg/dL	0.012	(0.000, 0.024)
Constant	−1.365***	(−1.873, −0.858)

^1^WAZ, *n* = 113; LAZ, *n* = 110. *,**,***Statistically significant: *P < 0.05, **P < 0.01, ***P < 0.001. FGF21, Fibroblast Growth Factor 21; LAZ, length-for-age *z* score; WAZ, weight-for-age *z* score.

^2^At enrollment.

The relation between FGF21 status and change in WAZ was similar in the 5-mo supplementation and postsupplementation periods. The linear mixed-effects model of 5-mo change in WAZ, which included both pre- and postsupplementation FGF21 data, had a nonsignificant interaction term for high FGF21 (*n* = 4) in the second period (**Table 3**). With an *n* of 4, the absence of significance cannot be distinguished from insufficient power. Therefore, a separate linear regression model of WAZ in the postsupplementation period was not created.

**FIGURE 3:**
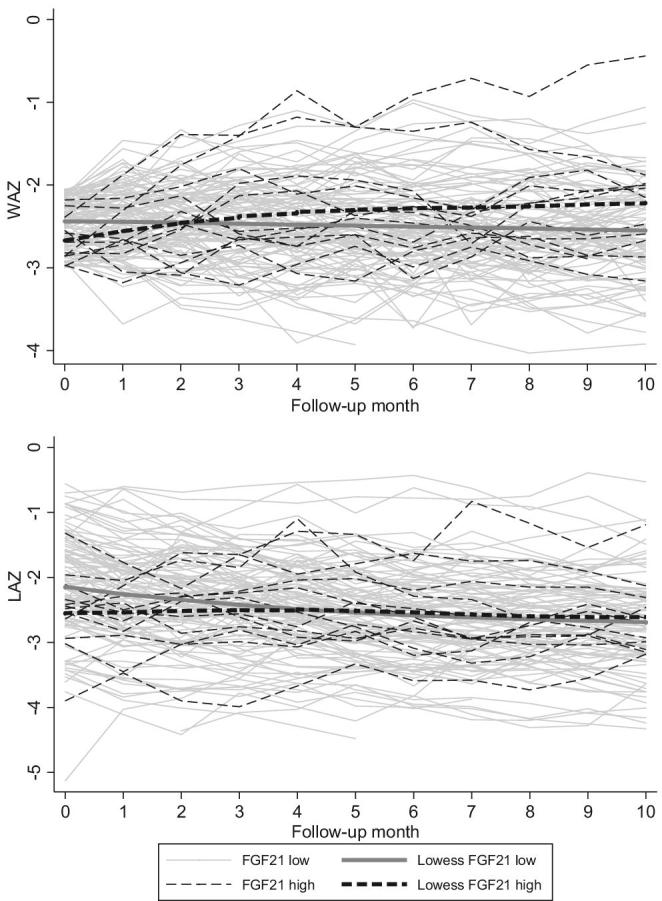
Ten-month anthropometry (WAZ and LAZ) trajectories and Lowess curves from 120 underweight children (WAZ = −3 to −2) grouped by high FGF21 (*n* = 12) and low FGF21 (*n* = 108) at enrollment. FGF21, Fibroblast Growth Factor 21; LAZ, length-for-age *z* score; WAZ, weight-for-age *z* score.

Monthly WAZ growth during the 5 mo of nutritional supplementation differed significantly by baseline FGF21 status based on the significant interaction term (*P* = 0.001) in the linear mixed-effects model containing all valid anthropometry data. During the 5 mo of nutritional supplementation, children with low baseline FGF21 concentrations did not gain or lose WAZ on average, whereas those with high FGF21 gained a mean 0.09 WAZ/mo (95% CI: 0.03, 0.14, *P* < 0.001; **Table 4**). This was equivalent to a gain of an additional 0.08 kg/mo among those with high baseline FGF21 in an age-adjusted model (**Supplemental Table 3**). Lowess curves (**Figure 3**) illustrate the individual and group WAZ trajectories.

### FGF21 and linear growth

Children who gained LAZ (*n* = 25) had a significantly higher mean baseline FGF21 concentration than those who lost LAZ (*n* = 89) over the 5 mo of supplementation (713.7 compared with 363.1 pg/mL, *P* = 0.0496). Plasma FGF21 concentrations at enrollment were significantly associated with 5-mo change in LAZ during supplementation in regression models (Table 2). Each 1-ng/mL increase in baseline plasma FGF21 was associated with a mean 0.19 (95% CI: 0.03, 0.36) more LAZ gain over 5 mo. High baseline FGF21 concentrations were significantly associated with 5-mo change in LAZ. Children with high FGF21 at enrollment gained a mean 0.54 LAZ (95% CI: 0.23, 0.84) more during supplementation than those with low FGF21.

The relation between FGF21 status and change in LAZ differed in the 5-mo supplementation and postsupplementation periods. The linear mixed-effects model of 5-mo change in LAZ, which included both pre- and postsupplementation FGF21 data, had a significant interaction term (*P* = 0.012; Table 3). High FGF21 values (*n* = 4) after supplementation were not associated with subsequent 5-mo change in LAZ in a linear regression model (Table 3).

Monthly linear growth during the 5 mo of nutritional supplementation differed significantly by baseline FGF21 status based on the significant interaction term (*P* < 0.001) in the linear mixed-effects model containing all valid anthropometry data. Adjustment for relevant micronutrient concentrations at baseline did not alter the significance of the interaction term in these models. During the 5 mo of nutritional supplementation, children with low baseline FGF21 concentrations lost a mean 0.09 LAZ/mo (95% CI: −0.11, −0.07, *P* < 0.001), whereas those with high FGF21 did not change LAZ (Table 4). This was equivalent to 0.3 cm more growth per month among those with high baseline FGF21 in an age-adjusted model (Supplemental Table 3). Lowess curves (Figure 3) illustrate the individual and group LAZ trajectories.

### Change in plasma FGF21 and growth during supplementation

During the 5 mo of supplementation, FGF21 concentrations decreased by a median of ‒62 pg/mL (IQR: −236, −34 pg/mL). Only 1 of the 12 children with high FGF21 (>1056.5 pg/mL) at baseline had high FGF21 at month 5. An additional 5 children had high FGF21 concentrations at month 5.

Children whose plasma FGF21 concentration increased (*n* = 42) during the 5 mo of supplementation lost a mean 0.22 more LAZ over the period than children whose plasma FGF21 concentration decreased (*n* = 72) (−0.55 compared with −0.33 LAZ, *P* = 0.0291). There was no significant difference in mean WAZ change between the 2 groups (−0.04 compared with 0.03 WAZ, *P* = 0.4484). Change in plasma FGF21 concentration during supplementation was negatively associated with 5-mo change in WAZ and LAZ in regression models (**Table 5**). Each 1-ng/mL increase in FGF21 concentration was associated with a mean loss of 0.48 WAZ (95% CI: −0.67, −0.29) and 0.31 LAZ (95% CI: −0.52, −0.11) during the 5 mo of supplementation.

**TABLE 5:** Multiple linear regression models of the relation between change in plasma FGF21 concentration and 5-mo change in WAZ and LAZ^1^

	Coefficient	(95% CI)
ΔWAZ		
WAZ^2^	−0.095	(−0.368, 0.178)
Change in FGF21, ng/mL	−0.481***	(−0.667, −0.294)
FGF21,^2^ ng/mL	−0.183	(−0.398, 0.032)
Ferritin,^2^ μg/L	0.001	(−0.001, 0.003)
Retinol,^2^ μg/dL	0.010	(−0.002, 0.022)
Constant	−0.477	(−1.221, 0.267)
ΔLAZ		
LAZ^2^	−0.341***	(−0.451, −0.232)
Change in FGF21, ng/mL	−0.313**	(−0.515, −0.110)
FGF21,^2^ ng/mL	−0.064	(−0.295, 0.168)
Ferritin,^2^ μg/L	−0.001	(−0.003, 0.002)
Retinol,^2^ μg/dL	0.009	(−0.004, 0.023)
Constant	−1.334***	(−1.747, −0.922)

^1^WAZ, *n* = 113; LAZ, *n* = 110. **,***Statistically significant: **P < 0.01, ***P < 0.001. FGF21, Fibroblast Growth Factor 21; LAZ, length-for-age *z* score; WAZ, weight-for-age *z* score.

^2^At enrollment.

**TABLE 3:** Linear mixed-effects models of the relations between FGF21 status (high compared with low) at baseline and postsupplementation with 5-mo change in WAZ and LAZ during and after supplementation (also shown: linear regression model of 5-mo change in LAZ during the postsupplementation period)^1^

	Coefficient	(95% CI)
ΔWAZ (*n* = 116)		
WAZ^2^	−0.048	(−0.186, 0.089)
Period	−0.039	(−0.159, 0.082)
High FGF21^3^	0.511***	(0.247, 0.774)
Period*FGF21 high^4^	−0.023	(−0.513, 0.467)
Ferritin,^2^ μg/L	0.001	(0.000, 0.003)
Retinol,^2^ μg/dL	0.003	(−0.005, 0.011)
Constant	−0.262	(−0.669, 0.144)
ΔLAZ (*n* = 115)		
LAZ^2^	−0.245***	(−0.320, −0.170)
Period	0.189**	(0.076, 0.303)
High FGF21^3^	0.565***	(0.310, 0.820)
Period*FGF21 high^4^	−0.596*	(−1.060, −0.132)
Ferritin,^2^ μg/L	−0.001	(−0.002, 0.001)
Retinol,^2^ μg/dL	0.013**	(0.005, 0.021)
Constant	−1.231**	(−1.481, −0.981)
Linear regression model of 5-mo change in LAZ in the		
postsupplementation period		
ΔLAZ (*n* = 96)		
LAZ at month 5	−0.113*	(−0.224, −0.003)
High FGF21^4^	0.003	(−0.293, 0.298)
Ferritin, μg/L (at month 5)	−0.001	(−0.003, 0.000)
Retinol, μg/dL (at month 5)	0.013**	(0.004, 0.021)
Constant	−0.696***	(−1.036, −0.356)

^1^*,**,***Statistically significant: *P < 0.05, **P < 0.01, ***P < 0.001. FGF21, Fibroblast Growth Factor 21; LAZ, length-for-age *z* score; WAZ, weight-for-age *z* score.

^2^At start of period.

^3^ *n* = 12 at enrollment.

^4^ *n* = 4 after supplementation.

**TABLE 4:** Linear mixed-effects models of the relations between month of follow-up and WAZ and LAZ, grouped by baseline FGF21 status, during 5 mo of nutritional supplementation^1^

	Low baseline FGF21 (*n* = 106)		High baseline FGF21 (*n* = 11)	
	Coefficient	(95% CI)	Coefficient	(95% CI)
WAZ^2^				
Month	−0.008	(−0.025, 0.008)	0.087**	(0.031, 0.144)
Ferritin,^3^ μg/L	0.001	(−0.001, 0.002)	0.014	(−0.002, 0.030)
Retinol,^3^ μg/dL	0.008	(−0.003, 0.018)	0.021	(−0.011, 0.053)
Constant	−2.634***	(−2.889, −2.378)	−3.251***	(−3.797, −2.705)
LAZ^4^				
Month	−0.086***	(−0.105, −0.067)	0.031	(−0.016, 0.077)
Ferritin,^3^ μg/L	0.003	(−0.001, 0.006)	−0.018	(−0.044, 0.009)
Retinol,^3^ μg/dL	0.012	(−0.008, 0.033)	0.021	(−0.034, 0.075)
Constant	−2.517***	(−2.994, −2.041)	−2.435***	(−3.342, −1.528)

^1^**,***Statistically significant: **P < 0.01, ***P < 0.001. FGF21, Fibroblast Growth Factor 21; LAZ, length-for-age *z* score; WAZ, weight-for-age *z* score.

^2^Interaction term *P* = 0.001.

^3^At enrollment.

^4^Interaction term *P* < 0.001.

### Environmental enteropathy and FGF21 status

The median fecal AAT concentration at enrollment was 0.268 mg/g among the 102 children with valid results (IQR: 0.163–0.673 mg/g; Table 1), with a highly skewed distribution (**Supplemental Figure 1**). The mean baseline AAT concentration was not significantly different between children with high and low baseline FGF21 concentrations (0.642 and 0.515 mg/g, respectively, *P* = 0.6189), and similar findings were observed when the values for AAT were log transformed.

## Discussion

In this sample of 120 moderately underweight children in Bangladesh, those with high baseline FGF21 demonstrated significant improvements in age-standardized weight and maintained linear growth during 5 mo of nutritional supplementation. In contrast, children with low baseline FGF21 experienced continued declines in age-standardized length and did not experience changes in weight during nutritional supplementation. These findings were independent of anthropometry, inflammation, and micronutrient status at enrollment, and are consistent with the hypothesis that high FGF21 concentrations may be useful as a biomarker to identify a priori which children are likely to grow in response to nutritional supplementation.

High FGF21 identified a small group of children who gained weight while receiving nutritional supplementation. The cohort was enrolled based on poor weight status, and those identified as having high baseline FGF21 improved their weight during supplementation to a greater degree than those with low FGF21 concentrations. The absence of improved linear growth among children with high baseline FGF21 is not surprising, because feeding interventions often fail to normalize linear growth (4). That these children did not become more stunted on average during supplementation is notable given that growth faltering in South Asian children generally worsens between 6 and 24 mo of age (32). Children with low baseline FGF21 became more stunted, growing on average ∼1.5 cm less and gaining nearly half a kilogram less over the 5 mo of supplementation. Therefore, our data suggest that FGF21 may be useful as a biomarker to identify which children are likely to grow in response to nutritional supplementation while also identifying those children in whom alternative interventions are likely to be required to improve growth. Environmental enteropathy, as measured by fecal concentrations of AAT, did not differ significantly by FGF21 status, and alterations in gut function and the gut microbiome may also have influenced growth in the cohort. Unfortunately, no interventions have been proven to effectively prevent or treat environmental enteropathy beyond transient improvements in gut function markers (33–36).

FGF21 concentrations are hypothesized to be part of the late adaptive response to starvation, as serum FGF21 increases in healthy human adults after 7–10 d of fasting (16, 37). Protein deprivation in particular appears to drive increases in circulating FGF21, as plasma concentrations in healthy adults increased markedly after 28 d of a low-protein diet but did not change in controls who were not protein restricted (14). Similarly, a small study in adults observed 6-fold higher plasma FGF21 concentrations in a group randomly assigned to receive a 5-d diet with 10% protein content than in the group who received a 25%-protein diet (15). The children with high FGF21 concentrations in our study had concentrations greatly exceeding those reported from healthy adults after a 10-d fast (16), suggesting that their high FGF21 concentrations may have been driven by sustained caloric and protein deprivation. There are limited published data on FGF21 concentrations in healthy children, and concentrations observed in this Bangladeshi cohort were much higher than those observed in a previous study of Chilean infants born preterm and full term and measured at birth and 6 mo of age (17). The importance of dietary protein for child growth is well documented and the typical infant diet after weaning from exclusive breastfeeding in Bangladesh often provides insufficient protein. The finding that FGF21 concentrations were positively associated with growth is somewhat counterintuitive given that both experimental and clinical studies have observed FGF21 to be positively associated with GH resistance and, thus, poor growth. However, it is plausible that nutritional supplementation of the type used in this study may have contributed to decline in FGF21 concentrations among children with high baseline concentrations by treating deficiencies in protein and macronutrient intake. High circulating FGF21 concentrations may be useful in identifying a subset of children whose poor growth status is in large part attributable to sustained caloric/protein deprivation, and who may therefore be more likely to grow in response to nutritional supplementation.

In all, 6 children had high FGF21 concentrations after 5 mo of supplementation. These children had significantly lower retinol and lower CRP concentrations after supplementation than children whose FGF21 concentrations were low. Interestingly, FGF21 concentration was negatively correlated with plasma retinol and positively correlated with AGP after supplementation. Both AGP and CRP are acute-phase proteins, but whereas CRP concentrations decline rapidly after infection, AGP falls off more slowly. That there were consistent correlations between FGF21 concentrations and both retinol and AGP at 2 different time points suggests that both chronic systemic inflammation and vitamin A insufficiency may influence FGF21 in addition to dietary protein. However, it is unclear why CRP concentrations were lower in the children with high FGF21 at month 5. Other factors, such as infection and intestinal injury, may also influence FGF21 and the present study may not provide sufficient discrimination to detect these associations.

This study is the first, to our knowledge, to evaluate the association between FGF21 and growth responsiveness during nutritional supplementation in underweight children. Previous pediatric studies have reported that plasma FGF21 is negatively associated with linear growth in preterm infants. For example, a study of very preterm infants reported a negative association between average FGF21 concentrations over the first 5 postnatal weeks and concurrent change in LAZ (13), suggesting that early elevated FGF21 concentrations are associated with postnatal growth failure. Another study reported a negative correlation between LAZ change from 6 to 12 mo of age and concurrent FGF21 change in a small sample of preterm infants (17). Consistent with these observations, in the present study, the 5-mo changes in both WAZ and LAZ were negatively associated with concurrent change in FGF21 concentration.

This study leveraged a well-designed and implemented cohort and had several notable strengths. Children were enrolled from a single setting using a well-defined recruitment protocol and rigorous inclusion and exclusion criteria established by the MAL-ED consortium. The high population density, poor sanitation, and low socioeconomic status of the Bauniabadh area are representative of a typical urban slum in Dhaka and are similar to others in South Asia (21). A unique strength in this study is that children were visited several times each week to collect highly detailed surveillance information on the incidence of diarrhea, respiratory disease, and other morbidity events. The detailed morbidity data enabled the exclusion of stool samples with close proximity to diarrhea symptoms or intestinal permeability testing that would have potentially diluted AAT concentrations (29). In addition, the use of linear mixed-effects models allowed for utilization of monthly anthropometry data, facilitated comparison of FGF21 and growth relations in the supplemented and nonsupplemented periods, and adequately accounted for correlated anthropometric data coming from the same individuals.

There were a few important limitations to this study. Children in the study were from a narrow age range and were restricted to those with WAZ between −3 and −2, limiting the generalizability of our conclusions. Useful data on gestational age, birth weight, and birth length were not possible to obtain because children were recruited and enrolled at a minimum age of 6 mo. Although a child's gestational age and size at birth may influence circulating FGF21 concentrations, these data were not available. Data on parity, plurality, and size of the parents were not available; however, there is no evidence to support a priori a relation between parity, plurality, or the size of parents and FGF21 concentrations in children. Owing to our choice of cutoff, there were relatively few children (10%) with high baseline concentrations of FGF21 and even fewer (5%) after supplementation. Therefore, results must be interpreted cautiously and tested in other populations. Owing to limited sample volumes, it was not possible to repeat FGF21 ELISAs using dilution where results greatly exceeded the absorbance of the highest standard. The intra-assay CV was 7.7% for the 226 samples within the detectable range of the assay (i.e., not those with assigned values). Some of this variability was likely because wavelength correction, used to correct for optical imperfections in the ELISA plate, was not performed. The timing of blood draws was not standardized and it is unclear whether diurnal cycle or proximity to meals influences FGF21 concentrations in children. However, FGF21 concentrations were relatively unaffected by time of day or standardized food intake over 24 h in 5 adults tested after a 25-h fast (37). Because there are limited child data available on endogenous FGF21 variability, it could have been useful to measure plasma FGF21 concentrations twice within an interval of 1–2 wk before supplementation in order to confirm the baseline FGF21 concentrations observed in the cohort.

Our evaluation of whether environmental enteropathy is associated with concentrations of FGF21 was limited by the small number of children with high FGF21 in the sample, and the sole reliance on AAT as a measure of environmental enteropathy. AAT describes elevated intestinal permeability, which is just one aspect of the range of functional changes thought to underlie the relation between environmental enteropathy and poor linear growth (38, 39). AAT may be less sensitive to small gaps in mucosal integrity owing to its large size than smaller molecules such as urinary lactulose. Without simultaneous plasma measurements, fecal AAT concentrations may provide a less reliable estimate of intestinal permeability, given the variability of plasma concentrations (40).

In summary, in a cohort of moderately underweight children, the subset with high initial FGF21 concentrations was most likely to respond to nutritional supplementation of the type used in this study, demonstrating improvement in WAZ. Although average LAZ remained relatively unchanged in children with high baseline FGF21, LAZ declined considerably in similarly underweight peers with low baseline FGF21. Changes in FGF21 concentrations were negatively associated with concurrent changes in children's LAZ and WAZ. FGF21 may identify undernourished children who are more and less likely to be growth responsive to nutritional supplementation. FGF21 and environmental enteropathy markers may be especially useful for improving the targeted treatment and prevention of poor growth in children.

## Supplementary Material

Supplemental File

## Notes

Supported by Bill & Melinda Gates Foundation: Environmental Enteric Dysfunction Planning Grant OPP1096963 (to JLW); Bangladesh Environmental Enteric Dysfunction Study; The Interactions of Malnutrition & Enteric Infections: Consequences for Child Health and Development (MAL-ED) study; the University of Washington Thomas Francis, Jr. Global Health Fellowship; and PATH. TA, RH, and MM received travel support from the funder (Bill & Melinda Gates Foundation) to attend the meeting of the project (MAL-ED) from which the FGF21 work has evolved. MBA received travel funding from the University of Washington Thomas Francis, Jr. Global Health Fellowship, Department of Epidemiology Conference Travel Award, Graduate and Professional Student Senate Travel Grant Award, and Graduate School Fund for Excellence and Innovation Travel Grant Award.

Author disclosures: MBA, BAR, MM, TA, RH, MAG, GCJ-S, DMD, JMS, and JLW, no conflicts of interest.

The funders had no role in the study design, data collection and analysis, decision to publish, or preparation of the manuscript.

Supplemental Methods, Supplemental Tables 1–3 and Supplemental Figure 1 are available from the “Supplementary data” link in the online posting of the article and from the same link in the online table of contents at https://academic.oup.com/cdn/.

Abbreviations used: AAT, α-1-antitrypsin; AGP, α-1-acid glycoprotein; CRP, C-reactive protein; ERC, Ethical Review Committee; FGF21, Fibroblast Growth Factor 21; GH, growth hormone; icddr,b, International Centre for Diarrhoeal Disease Research, Bangladesh; IGF-I, insulin-like-growth factor I; LAZ, length-for-age *z* score; MAL-ED, The Interactions of Malnutrition & Enteric Infections: Consequences for Child Health and Development; SAM, severe acute malnutrition; WAZ, weight-for-age *z* score; WLZ, weight-for-length *z* score.
