# Supplementary material for: Plasma Fibroblast Growth Factor 21 Is Associated with Subsequent Growth in a Cohort of Underweight Children in Bangladesh
Source: Curr Dev Nutr. 2019 Mar 30;3(5):nzz024. doi: 10.1093/cdn/nzz024 (PMC6511337; doi:10.1093/cdn/nzz024)
Supplement: Supplemental File [file nzz024_supplemental_file.docx]

**Comment regarding sample size calculations:** A sample size of 109 would have provided 80% power to detect true slopes of ‒0.004 or 0.004 for the line obtained by regressing six-month change in length-for-age z-score (LAZ) against concurrent change in Fibroblast Growth Factor 21 (FGF21); however, the sample was increased by 10% (to 120 children) to guard against any missing values. The standard deviations (SDs) and correlation coefficient used in the power calculation came from a sample of Chilean infants, whose anthropometry and FGF21 levels were assessed at six-month intervals (1). The SD for six-month change in LAZ was 0.56, and the correlation coefficient between change in LAZ and concurrent change in FGF21 was ‒0.19. The SD for change in FGF21 was not presented in the publication, so the SD was extrapolated by dividing the interquartile range (IQR) of six-month change in FGF21 by 1.35, as the IQR is approximately 1.35 times the SD in normally distributed data. As the IQR was not available for the pooled sample of 80 children, the SD calculation was repeated for the two groups of 20 preterm children who were small or average for gestational age at birth (IQRs of 68.5 and 32.2 pg/ml, respectively), and averaged to get an SD of 37.3 pg/ml.

**References**

1. Mericq V, Luca F, Hern M, Hernandez M, Peña V, Rossel K, Garcia M, Avila A, Cavada G, Iñiguez G. Serum fibroblast growth factor 21 levels are inversely associated with growth rates in infancy. Horm Res Paediatr. 2014;82:324‒31.

| **SUPPLEMENTAL TABLE 1** Pearson correlations between FGF21 concentrations and potential cofactors at enrollment and at five-month follow-up | | | | | | | | | |
| --- | --- | --- | --- | --- | --- | --- | --- | --- | --- |
|  |  | | **Baseline** | | |  | **Month five** | | |
|  |  | **n** | | **rho** | **p-value** |  | **n** | **rho** | **p-value** |
| **Age (days)** |  | 120 | | ‒0.0781 | 0.3964 |  | 116 | ‒0.1579 | 0.0905 |
|  |  |  | |  |  |  |  |  |  |
| **Anthropometry** | WAZ | 120 | | ‒0.2103 | 0.0211 |  | 116 | ‒0.3744 | 0.0000 |
|  | LAZ | 119 | | ‒0.1464 | 0.1122 |  | 115 | ‒0.2686 | 0.0037 |
|  | WLZ | 119 | | 0.0505 | 0.5856 |  | 115 | ‒0.1957 | 0.0361 |
|  |  |  | |  |  |  |  |  |  |
| **Stool marker** | AAT (mg/g) | 102 | | ‒0.0218 | 0.8277 |  | 102 | ‒0.0460 | 0.6462 |
| **Plasma micronutrient concentrations** |  |  | |  |  |  |  |  |  |
|  | Ferritin (μg/L) | 118 | | ‒0.0846 | 0.3627 |  | 114 | ‒0.0228 | 0.8097 |
|  | Hemoglobin | 100 | | ‒0.0217 | 0.83 |  | 92 | 0.0876 | 0.4065 |
|  | Retinol (μg/dl) | 117 | | ‒0.3365 | 0.0002 |  | 116 | ‒0.2422 | 0.0088 |
|  | Vitamin D (nmol/L) | 116 | | ‒0.0648 | 0.4897 |  | 114 | 0.1384 | 0.1421 |
|  | Zinc (mg/L) | 114 | | ‒0.1356 | 0.1504 |  | 110 | ‒0.1343 | 0.162 |
|  |  |  | |  |  |  |  |  |  |
| **Plasma inflammatory markers** | AGP (mg/dl) | 116 | | 0.2657 | 0.0039 |  | 114 | 0.2779 | 0.0028 |
|  | CRP (mg/dl) | 117 | | 0.1324 | 0.1548 |  | 114 | ‒0.0786 | 0.4056 |
|  |  |  |  |  |  |  |  |  |  |

*Abbreviations:* AAT, alpha-1-antitrypsin; AGP, alpha-1-acid glycoprotein; CRP, C-reactive protein; FGF21, Fibroblast Growth Factor 21; LAZ, length-for-age z-score; WAZ, weight-for-age z-score; WLZ, weight-for-length z-score.

Observations missing for more than 5% of participants: AAT, 102 valid observations; hemoglobin, 100 observations; zinc, 114 observations.

**SUPPLEMENTAL TABLE 2** Baseline characteristics of participants by baseline FGF21 status

|  |  | **Low baseline FGF21  (n = 108)** | | **High baseline FGF21  (n = 12)** | |
| --- | --- | --- | --- | --- | --- |
|  |  | **n (%) or median (IQR)** | | **n (%) or median (IQR)** | |
| **Female** |  | 59 | (54.6) | 8 | (66.7) |
| **Child parity** |  | 2 | (1, 2) | 1 | (1, 1.5) |
| **Age (months)** |  | 9.1 | (7.3, 11.1) | 9.7 | (7.3, 10.8) |
| **Household income** |  | 7,000 | (5,000, 8,500) | 7,000 | (5,500, 10,000) |
|  |  |  |  |  |  |
| **Mother** | Never attended school | 20 | (18.5) | 2 | (16.7) |
|  | Years of education | 4 | (2, 6) | 4 | (2, 6.5) |
|  | Age (years) | 23 | (20, 27) | 21 | (20, 25) |
|  |  |  |  |  |  |
| **Anthropometry** | LAZ | ‒2.13 | (‒2.61, ‒1.58) | ‒2.46 | (‒2.79, ‒2.22) |
|  | Stunted | 61 | (57.0) | 10 | (83.3) |
|  | WAZ | ‒2.39 | (‒2.71, ‒2.20) | ‒2.67 | (‒2.86, ‒2.47) |
|  | ZHC | ‒1.85 | (‒2.56, ‒1.25) | ‒1.9 | (‒2.62, ‒1.52) |
|  | WLZ | ‒1.70 | (‒2.17, ‒1.28) | ‒1.64 | (‒1.71, ‒1.10) |
|  | Wasted | 35 | (32.7) | 2 | (16.6) |
|  |  |  |  |  |  |
| **Stool marker** | AAT (mg/g) | 0.26 | (0.16, 0.67) | 0.38 | (0.21, 0.60) |
|  |  |  |  |  |  |
| **Plasma micronutrient concentrations** | Ferritin (μg/L) | 23.9 | (10.9, 45.2) | 19.9 | (14.8, 31.0) |
|  | Retinol (μg/dl) | 21.0 | (17.5, 25.7) | 13.3 | (10.8, 17.7) |
|  | Vitamin D (nmol/L) | 66.6 | (52.4, 81.4) | 57.1 | (50.4, 69.9) |
|  | Zinc (mg/L) | 0.74 | (0.65, 0.83) | 0.74 | (0.65, 0.79) |
|  |  |  |  |  |  |
| **Plasma inflammatory markers** | AGP (mg/dl) | 99.1 | (74.4, 126.0) | 121.0 | (73.8, 163.0) |
|  | CRP (mg/dl) | 1.2 | (0.4, 3.6) | 1.8 | (0.4, 4.4) |

*Abbreviations:* AAT, alpha-1-antitrypsin; AGP, alpha-1-acid glycoprotein; CRP, C-reactive protein; FGF21, Fibroblast Growth Factor 21; IQR, interquartile range; LAZ, length-for-age z-score; WAZ, weight-for-age z-score; WLZ, weight-for-length z-score.

Observations missing for more than 5% of participants: AAT, 102 valid observations; hemoglobin, 100 observations; zinc, 114 observations.

| **SUPPLEMENTAL TABLE 3** High FGF21 and anthropometry (metric scale) during five months of supplementation | | | |
| --- | --- | --- | --- |
| **Outcome** | | **Coefficient** | **(95% CI)** |
| **Weight (kg)** | Month of follow-up | 0.17^‡^ | (0.16, 0.19) |
|  | Baseline weight | 1.03^‡^ | (0.94, 1.13) |
|  | FGF21 high | 0.06 | (‒0.11, 0.24) |
|  | Month*FGF21 high | 0.08^‡^ | (0.03, 0.12) |
|  | Ferritin (μg/L) | 0.00 | (0, 0) |
|  | Retinol (μg/dl) | 0.01 | (0, 0.01) |
|  | Baseline age | 0.00 | (0, 0) |
|  | Constant | ‒0.26 | (‒0.81, 0.29) |
|  |  |  |  |
| **Height (cm)** | Month of follow-up | 0.9^‡^ | (0.846, 0.937) |
|  | Baseline height | 0.9^‡^ | (0.814, 0.941) |
|  | FGF21 high | ‒0.1 | (‒0.643, 0.5) |
|  | Month*FGF21 high | 0.3^‡^ | (0.133, 0.425) |
|  | Ferritin (μg/L) | 0.0 | (‒0.005, 0.002) |
|  | Retinol (μg/dl) | 0.0 | (‒0.005, 0.034) |
|  | Baseline age | 0.0 | (‒0.002, 0.004) |
|  | Constant | 0.9^‡^ | (0.846, 0.937) |

*Abbreviations:* CI, confidence interval; FGF21, Fibroblast Growth Factor 21.

^‡^ p <0.001.

0

10

20

30

40

50

Percent

0

.5

1

1.5

2

2.5

Fecal AAT concentration (mg/g)

**SUPPLEMENTAL FIGURE 1** Fecal alpha-1-antitrypsin (AAT) concentrations at enrollment (N = 102).
